# Patients with Chronic Liver Disease under Surveillance for Hepatocellular Carcinoma Have a Favorable Long-Term Outcome for Pancreatic Cancer Due to Early Diagnosis and High Resection Rate

**DOI:** 10.3390/cancers15030561

**Published:** 2023-01-17

**Authors:** Teru Kumagi, Takashi Terao, Taira Kuroda, Mitsuhito Koizumi, Yoshiki Imamura, Yoshinori Ohno, Tomoyuki Yokota, Nobuaki Azemoto, Kazuhiro Uesugi, Yoshiyasu Kisaka, Yoshinori Tanaka, Naozumi Shibata, Hideki Miyata, Teruki Miyake, Yoichi Hiasa

**Affiliations:** 1 Gastroenterology and Metabology, Ehime University Graduate School of Medicine, To-on 791-0295, Japan; 2 Postgraduate Medical Education Center, Ehime University Hospital, To-on 791-0295, Japan; 3 Gastroenterology, National Hospital Organization Shikoku Cancer Center, Matsuyama 791-0280, Japan; 4 Gastroenterology, Ehime Prefectural Central Hospital, Matsuyama 790-0024, Japan; 5 Center for Liver-Biliary-Pancreatic Diseases, Matsuyama Red Cross Hospital, Matsuyama 790-8524, Japan; 6 Gastroenterology, Uwajima Municipal Hospital, Uwajima 798-8510, Japan; 7 Gastroenterology, Matsuyama Shimin Hospital, Matsuyama 790-0067, Japan; 8 Internal Medicine, Niihama Prefectural Hospital, Niihama 792-0042, Japan

**Keywords:** pancreatic cancer, chronic liver disease, early diagnosis, surgical resection, long-term outcome

## Abstract

**Simple Summary:**

Patients with pancreatic cancer are often diagnosed at an advanced stage. However, the incidental detection of pancreatic duct dilatation during surveillance for hepatocellular carcinoma often leads to the early diagnosis of pancreatic cancer in patients with viral hepatitis-related chronic liver disease. While pancreatic cancer can be diagnosed early, patients may have advanced chronic liver disease that precludes surgical resection, and the long-term prognosis is unknown for these patients. This study aimed to determine the long-term prognosis of pancreatic cancer patients with viral hepatitis-related chronic liver disease. We found that patients with viral hepatitis-related chronic liver disease under surveillance for hepatocellular carcinoma have a favorable long-term outcome for pancreatic cancer due to earlier diagnosis at earlier stages when resection is more feasible.

**Abstract:**

Patients with viral hepatitis-related chronic liver disease (CLD) under surveillance for hepatocellular carcinoma (HCC) are often diagnosed with pancreatic cancer (PC) at an early stage. However, the long-term outcomes of these patients are unclear. We aimed to clarify the long-term outcomes of patients with PC with viral hepatitis-related CLD using a chart review. Data collection included the Union for International Cancer Control (UICC) stage at PC diagnosis, hepatitis B virus and hepatitis C virus status, and long-term outcomes. The distribution of the entire cohort (*N* = 552) was as follows: early stage (UICC 0-IB; *n* = 52, 9.5%) and non-early stages (UICC IIA-IV; *n* = 500, 90.5%). At diagnosis, the HCC surveillance group (*n* = 18) had more patients in the early stages than the non-surveillance group (*n* = 534) (50% vs. 8.0%), leading to a higher indication rate for surgical resection (72.2% vs. 29.8%) and a longer median survival time (19.0 months vs. 9.9 months). We confirmed that patients with viral hepatitis-related CLD under HCC surveillance were diagnosed with PC at an early stage. Because of the higher indication rate for surgical resection in these patients, they had favorable long-term outcomes for PC.

## 1. Introduction

Pancreatic cancer (PC) is one of the most common cancers worldwide but has the worst prognosis, with a 5-year survival rate of 5–10% [1]. This is primarily due to the lack of an algorithm for diagnosing PC at early stages, leaving only 20–30% of PC patients undergoing surgical resection [2,3]. To improve the long-term outcomes of PC, more patients need to be diagnosed early, preferably within stage IIA (without lymph node and distant metastasis) of the Union for International Cancer Control (UICC). Patients at this stage are better candidates for surgical resection. A thorough investigation of indirect features in the pancreas, such as a dilated pancreatic duct or a cystic lesion detected on abdominal imaging, can facilitate an early diagnosis of PC [4,5]. Recent studies of high-risk patients, including familial PC and CDKN2A pathogenic variant carriers, have shown that the participation in longitudinal surveillance protocols increases the probability of being diagnosed with early PC and prolongs survival [6,7].

In view of the high incidence of hepatocellular carcinoma (HCC) in patients with advanced liver fibrosis, periodic surveillance imaging is strongly recommended worldwide for these patients [8,9,10]. We recently studied whether the HCC surveillance group (patients with HBV- and HCV-related chronic liver disease under surveillance for HCC) was diagnosed with PC at an early stage [11]. In this study, the HCC surveillance group had a significantly higher rate of patients with PC at a very early stage (UICC stage 0) compared to the non-surveillance group, owing to incidental abnormal findings in the pancreas on imaging studies. Notably, all the patients diagnosed with stage 0 disease had pancreatic duct dilatation incidentally detected on abdominal images. Our results strongly support the importance of further investigation of indirect features detected incidentally in the pancreas, regardless of the reasons for imaging. However, only scattered studies report on the long-term outcomes of PC patients diagnosed early [12,13,14,15]. Moreover, these patients may not have a good prognosis because some may have liver dysfunction, which may alter the management of PC. Indeed, in a daily clinical setting, we may encounter PC patients with decreased liver function due to liver cirrhosis. Various medical interventions are limited in these patients, and surgical resection is particularly problematic [12,13].

In this regard, the Ehime Pancreato-Cholangiology (EPOCH) Study Group conducted a longitudinal study to clarify the long-term outcomes of PC patients diagnosed during HCC surveillance.

## 2. Materials and Methods

This retrospective study examined data from 552 patients who were diagnosed with PC between January 2011 and December 2013 at Ehime University Hospital and its affiliated centers (EPOCH Study Group) after excluding patients with unknown viral hepatitis status (hepatitis B surface antigen [HBsAg] and anti-HCV antibodies). We surveyed data on age, sex, the trigger of PC diagnosis, liver-related blood tests [platelet counts, aspartate aminotransferase (AST), alanine aminotransferase (ALT), total bilirubin, and albumin], and UICC stage (7th edition) [16]. All data were collected at the time of PC diagnosis (or before PC treatment for patients requiring biliary drainage due to obstructive jaundice). Data regarding patient outcomes (survival, lost to follow-up, and death) were collected as of May 2017.

PC diagnosis was based on tumor markers, abdominal imaging, and/or histological findings, as described elsewhere [3]. The staging of PC was determined according to the clinical stage, except for stage 0, which was confirmed pathologically [11]. Since a nationwide survey of PC revealed significant differences in 5-year survival between stage IB (59.7%) and stage IIA (30.2%) [2], stages 0–IB were defined as early stages in this study, while stages IIA–IV were defined as non-early stages.

In this study, liver biopsy was considered the gold standard for diagnosing CLD. The aspartate aminotransferase-to-platelet ratio index (APRI) was calculated in patients with CLD. However, the final decision on the interval of HCC surveillance is made by individual hepatologists. Abdominal imaging (ultrasound, CT, MRI) and tumor markers analysis (AFP, DCP) were generally performed every three to four months in cirrhosis patients and every six months in non-cirrhosis patients, according to the guideline recommendations [10].

Data are reported as mean ± standard deviation or number and percentage. Intergroup comparisons were performed using the chi-squared test. Outcomes were analyzed using the Kaplan–Meier method and Cox proportional hazards regression. Differences in survival analyses were determined using the log-rank test. The differences were considered statistically significant at a two-tailed *p*-value < 0.05. All statistical analyses were performed using JMP software (version 13; SAS Institute, Cary, NC, USA). To ensure the anonymity of patient data, the data were stored in a secure database, and the patients were numerically coded. The study protocol complied with the ethical guidelines of the Declaration of Helsinki and was approved by the ethics committee of Ehime University Graduate School of Medicine (1204066). The requirement for written consent was waived owing to the study’s retrospective nature.

## 3. Results

### 3.1. Baseline Demographics at PC Diagnosis

The mean age of the entire cohort was 71.3 ± 10.2 years. Males were 54.5% of the patients. There was no difference in age or gender between the HCC surveillance and the non-surveillance groups (Table 1).

The diagnostic triggers for PC in the HCC surveillance group (*n* = 18) were symptoms (*n* = 5, 27.8%) and incidental findings in the pancreas (*n* = 13, 72.2%) [HCC surveillance (*n* = 9) and other diseases (*n* = 4)]. The diagnostic triggers for PC in the non-surveillance group (*n* = 534) were symptoms (*n* = 428, 80.1%), incidental findings in the pancreas (*n* = 42, 7.9%) [health checkup (*n* = 11), during follow-up for IPMN (*n* = 6), and other conditions, mainly pre-/post-operative screening for cancer (*n* = 25)], diabetes-related (*n* = 17, 3.2%) [new onset diabetes or worsening of diabetes without symptoms (*n* = 14), screening for well-controlled diabetes (*n* = 3)], and unknown or unclassifiable (*n* = 47). The HCC surveillance group had more patients with incidental findings in the pancreas than the non-surveillance group (*p* < 0.0001). In addition, platelet counts were lower in the HCC surveillance group than in the non-surveillance group (*p* = 0.0103). However, there were no differences in other parameters between the two groups. As for transaminase (AST and ALT) and albumin, patients with total bilirubin levels above 2.0 mg/dL (*n* = 268) were excluded from the analysis to minimize the influence of obstructive jaundice due to PC.

### 3.2. UICC Stages and Treatments in Patients with PC

The distribution of the UICC stage of the entire cohort (*N* = 552) was as follows: 0 (*n* = 7, 1.3%), IA–B (*n* = 45, 8.2%), IIA–B (*n* = 171, 31.2%), III (*n* = 79, 14.3%), and IV (*n* = 250, 45.1%). The HCC surveillance group (*n* = 18) had 9 patients (50%) in the early stages, whereas the non-surveillance group (*n* = 534) had only 43 patients (8.0%). When compared to the non-surveillance group, the HCC surveillance group had more patients diagnosed with PC at earlier stages (*p* < 0.0001) (Table 2). The HCC surveillance group had fewer patients with pancreatic head involvement; however, this finding was not statistically significant (44.4% vs. 57.1%, *p* = 0.2859). The entire cohort had 172 patients who underwent surgical resection, which resulted in R0 resection (*n* = 121), non-R0 resection (*n* = 25), and unknown (*n* = 26). As for the R0 resection, the circumferential resection margin with the 1 mm rule (R0CRM-/+) was not reported but the actual distance was reported in most of the cases. The indication rate for surgical resection in the HCC surveillance group (*n* = 13, 72.2%) was significantly higher than that in the non-surveillance group (*n* = 210, 39.3%) (*p* = 0.0067).

With regards to the patients indicated for resection in the HCC surveillance group (Table 3), seven (53.8%) had an APRI score greater than 0.7, which suggests significant hepatic fibrosis. In comparison, three (23.1%) had an APRI score greater than 1.0, suggesting cirrhosis [17,18]. None of the nine patients had pancreatic head involvement in the early stages of the disease. Four patients did not opt for surgical resection for various reasons [stage IIA (*n* = 3), pancreatic head lesions (*n* = 3), APRI score of 0.7 to 1.0 (*n* = 3)] in addition to advanced age. However, none of them were contraindicated for advanced liver disease. Finally, nine patients underwent surgical resection.

### 3.3. Long-Term Outcomes of PC Patients

**Figure 1:**
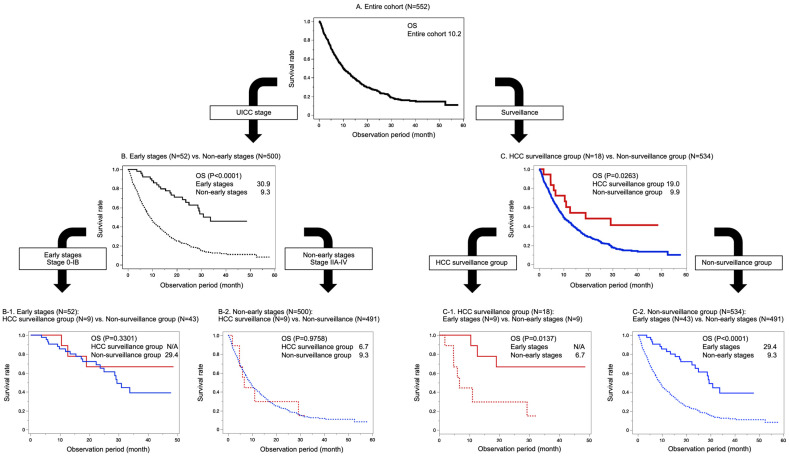
Long-term outcomes of pancreatic cancer patients (**A**). Comparison between the stages (**B**) and their corresponding surveillance groups (**B1**,**B2**). Comparison between the surveillance groups (**C**) and their corresponding stages (**C1**,**C2**). The lines represent the following: entire cohort (black bold), early stages (black solid), non-early stages (black dot), HCC surveillance group (red bold), non-surveillance group (blue bold), early stages/HCC surveillance group (red solid), early stages/non-surveillance group (blue solid), non-early stages/HCC surveillance group (red dot), and non-early stages/non-surveillance group (blue dot). OS, overall survival; UICC, Union for International Cancer Control; HCC, hepatocellular carcinoma.

The median overall survival (OS) of the entire cohort (*N* = 552) was 10.2 months (Figure 1A). When the UICC stage was compared, the median OS of the early stages (*n* = 52) was longer than that of the non-early stages (*n* = 500) (Figure 1B, 30.9 months vs. 9.3 months, *p* < 0.0001). However, the median OS of the early stages did not differ between the HCC surveillance group (*n* = 9) and the non-surveillance group (*n* = 43) (Figure 1B1, *p* = 0.3301). Similarly, the median OS of the non-early stages did not differ between the HCC surveillance group (*n* = 9) and the non-surveillance group (*n* = 491) (Figure 1B2, *p* = 0.9758). Additionally, when the mode of surveillance was compared, the median OS was significantly longer in the HCC surveillance group (*n* = 18) than in the non-surveillance group (*n* = 534) (Figure 1C, 19.0 months vs. 9.9 months, *p* < 0.0001). Furthermore, sub-analysis revealed that the early stages had a longer median OS than the non-early stages in the HCC surveillance group (Figure 1C1, *p* = 0.0137) and the non-surveillance group (Figure 1C2, *p* < 0.0001). Finally, despite having a history of HCC, there were no liver-related deaths among patients in the HCC surveillance group.

## 4. Discussion

In this study, we first confirmed that patients under surveillance for HCC are diagnosed with PC at an early stage because these patients are the only population undergoing periodic abdominal imaging. Additionally, these patients had favorable long-term outcomes for PC. However, the long-term outcomes did not differ when the stages (early and non-early) were compared between the HCC surveillance and the non-surveillance groups.

We present two novel findings in this study. First, we demonstrated that HCC surveillance for HBV- and HCV-related chronic liver disease significantly contributes to the early diagnosis of PC. This finding is not surprising, since we simply added collaborative institutes to our previously studied institutes [11]. The primary purpose of this study was to clarify the long-term outcomes of HCC surveillance.

Second, the HCC surveillance group not only was diagnosed with PC at earlier stages but also had a higher indication rate for surgical resection and favorable long-term outcomes. Surgical resection in patients with cirrhosis is challenging and is generally associated with various risk factors. Furthermore, the prognosis of PC patients following surgical resection, particularly pancreatoduodenectomy, is another concern. Several studies have recently shown that short- and long-term outcomes of pancreatoduodenectomy for PC are favorable when the patients are within Child–Pugh Class A and are properly managed [12,13,14]. Although we did not have any patients with pancreatic head involvement in the HCC surveillance group within the early stages, and the main reason for not undergoing surgical resection in our cohort was aging, careful attention should be paid to the selection of surgical resection in PC patients with liver cirrhosis.

Finally, the long-term outcomes of the HCC surveillance and non-surveillance groups did not differ when the UICC stages were matched (early stages vs. non-early stages), despite the HCC surveillance group being diagnosed with PC at early stages. This result strongly indicates that the prognosis is poor when patients are diagnosed at advanced stages, illustrating the significance of the early diagnosis of PC.

As this is a retrospective study, there are some limitations to our study. First, our data are dated, and the analysis was conducted in 2017 when we did not have the UICC 8th edition. However, we believe that the results were not affected largely, since the idea of HCC surveillance and the recognition of extensive investigation for PC have not changed since then. Second, we did not have precise data on the imaging intervals for HCC surveillance, although each hepatologist likely followed the guideline. Obtaining retrospective serial data may facilitate the identification of novel findings that can be used to detect PC at early stages, even before the emergence of a dilated pancreatic duct or cyst [19]. Third, most of the resection cases had PC in the pancreatic tail. Further studies with more patients, including those with cancer in the pancreatic head, are needed to clarify the true impact of long-term outcomes. Forth, the idea of mesopancreatic excision, which improves local disease control and survival, was not widely implemented in those days [20]. Finally, our cohort did not include patients with liver failure that would alter the management of PC, for whom, particularly, surgical resection would be a contraindication, even when PC is diagnosed at stages when resection is more feasible. Thus, we could not clarify this in our cohort, presumably because the prevalence of PC in patients with HBV- and HCV-related chronic liver disease is low. However, a recent retrospective study involving 529 patients with chronic hepatic dysfunction, including 105 cirrhotic patients who underwent PD at high-volume institutions, indicated that portal hypertension, prothrombin activity <70%, serum AST > 50 IU/L, and APRI greater than 1.0, are associated with an increase in patient mortality [15].

## 5. Conclusions

We report that patients with HBV- and HCV-related chronic liver disease who were under surveillance for HCC had favorable long-term outcomes for PC because of the higher indication rate for surgical resection. However, to improve the prognosis of PC, indirect features of the pancreas that are incidentally discovered require extensive investigation, regardless of the initial detection clue.

## Figures and Tables

**Table 1:** Baseline demographics at PC diagnosis.

	HCC Surveillance Group (*n* = 18)	Non-Surveillance Group (*n* = 534)	*p*-Value
Age	71.4 ± 11.6	71.3 ± 10.2	0.0900
Sex (male/female)	12/6	289/245	0.2931
PC diagnosis (symptomatic vs. others)			<0.0001
Symptomatic (*n* = 433)	5 (27.8%)	428 (80.1%)	
DM-related (*n* = 17)	0	17 (3.2%)	
Health checkup (*n* = 11)	0	11 (2.1%)	
During HCC surveillance (*n* = 9)	9 (50%)	N/A	
IPMN follow-up (*n* = 6)	0	6 (1.1%)	
Other specific conditions (*n* = 29)	4 (22.2%)	25 (4.7%)	
Unknown/unclassifiable (*n* = 47)	0	47 (8.8%)	
Liver-related blood tests (jaundice case excluded from analysis)			
Platelet counts (×10^3^/μL)	173 ± 63	223 ± 77	0.0103
AST (IU/L)	32 ± 14	31 ± 30	0.9046
ALT (IU/L)	29 ± 16	31 ± 47	0.9053
Total bilirubin (mg/dL)	0.77 ± 0.51	0.67 ± 0.30	0.2899
Albumin (g/dL)	3.99 ± 0.52	3.77 ± 0.56	0.1458

PC, pancreatic cancer; HCC, hepatocellular carcinoma; AST, aspartate aminotransferase; ALT, alanine aminotransferase; UICC, Union for International Cancer Control.

**Table 2:** UICC stages and treatments in patients with PC.

	HCC Surveillance Group (*n* = 18)	Non-Surveillance Group (*n* = 534)	*p*-Value
UICC stages (early vs. non-early stages)			<0.0001
Early stages (0–IB, *n* = 52)	9 (50%)	43 (8.0%)	
Non-early stages (IIA–IV, *n* = 500)	9 (50%)	491 (91.9%)	
Pancreatic head involvement			0.2859
Yes	8 (44.4%)	305 (57.1%)	
No	10 (55.6%)	229 (42.9%)	
Indication for surgical resection	13 (72.2%)	210 (39.3%)	0.0067
Treatments			0.1325
Surgical resection ± chemotherapy	9 (50%)	174 (32.6%)	
Chemotherapy	6 (33.3%)	239 (44.8%)	
Palliative care alone	3 (16.7%)	110 (20.6%)	
Unknown	0	11 (2.1%)	
Resection status (R0 vs. others)			0.7200
R0 (*n* = 123)	7	116	
Non-R0 (*n* = 25)	0	25	
Unknown (*n* = 35)	2	33	

HCC, hepatocellular carcinoma; UICC, Union for International Cancer Control.

**Table 3:** Profile of the PC patients indicated for surgical resection in the HCC surveillance group.

Age	Sex	Liver Profile							Pancreatic Cancer Profile			
		Viral	Platelet (×10^3^/μL)	AST (IU/L)	APRI	T.Bil (mg/dl)	Alb (g/dl)	PT (%)	UICC Stage	Pancreatic Head Involvement	Treatment	Outcome (Months)
80	M	HCV	185	20	0.292	0.4	4.1	90	0	No	DP	Alive (40.6)
62	M	HBV	247	26	0.284	0.6	4.8	113	0	No	DP	Alive (43.7)
84	M	HCV	121	54	1.206	0.4	4.4	85	0	No	DP	Alive (48.7)
79	F	HBV	88	33	1.014	0.6	3.1	67	IA	No	DP	Alive (25.1)
81	M	HCV ^a^	116	65	1.514	1.1	3.7	82	IA	No	DP	Deceased (10.5)
80	F	HCV	163	45	0.746	0.4	3.8	N/A	IA	No	Palliative	Deceased (12.7)
70	M	HCV	97	24	0.669	1.0	4.5	103	IB	No	DP	Alive (31.3)
43	F	HBV	199	21	0.150	0.9	4.1	100	IB	No	DP	Alive (37.6)
66	M	HBV	209	32	0.414	0.4	4.2	118	IB	No	DP	Deceased (19.0)
78	M	HCV	103	30	0.787	0.4	4.5	107	IIA	Yes	CT	Alive (7.0)
75	F	HCV	178	28	0.425	0.5	4.0	97	IIA	Yes	CRT	Deceased (4.8)
78	F	HCV	297	89	0.810	1.0	3.2	36 ^b^	IIA	Yes	CRT	Deceased (6.7)
64	M	HCV	246	25	0.275	0.6	4.4	79	IIA	Yes	PD	Deceased (29.2)

PC, pancreatic cancer; HCC, hepatocellular carcinoma; AST, aspartate aminotransferase; APRI, AST-to-platelet ratio index; T.Bil, total bilirubin; Alb, albumin; PT, prothrombin time; UICC, Union for International Cancer Control; M, male; F, female; HBV, hepatitis B virus; HCV, hepatitis C virus; N/A, not available; DP, distal pancreatectomy; CT, chemotherapy; CRT, chemoradiation therapy; PD, pancreatoduodenectomy; a, History of HCC. b, On warfarin.

## Data Availability

Data presented are contained within the article. For additional information, datasets are also available from the corresponding author upon reasonable request.
